# Inconsistent diagnosis of acute malnutrition by weight-for-height and mid-upper arm circumference: contributors in 16 cross-sectional surveys from South Sudan, the Philippines, Chad, and Bangladesh

**DOI:** 10.1186/s12937-015-0074-4

**Published:** 2015-08-25

**Authors:** Dominique Roberfroid, Lieven Huybregts, Carl Lachat, France Vrijens, Patrick Kolsteren, Benjamin Guesdon

**Affiliations:** 1 Department of Public Health, Institute of Tropical Medicine, Antwerp, Belgium; 2 Belgian Health Care Knowledge Center, Brussels, Belgium; 3 International Food Policy Research Institute, Washington, DC USA; 4 Faculty of Bioscience Engineering, Ghent University, Ghent, Belgium; 5 Action Against Hunger, Paris, France

## Abstract

**Background:**

The two anthropometric indicators of acute malnutrition in children under 5 years, i.e. a Mid-Upper Arm Circumference < 125 mm (MUAC_125_) or a Weight-for-Height Z-score<−2 (WHZ_−2_), correlate poorly. We aimed at assessing the contribution of age, sex, stunting (Height-for-Age HAZ<−2), and low sitting-standing height ratio Z-score (SSRZ in the 1st tertile of the study population, called hereafter ‘longer legs’) to this diagnosis discrepancy.

**Methods:**

Data from 16 cross-sectional nutritional surveys carried out by Action Against Hunger International in South Sudan, the Philippines, Chad, and Bangladesh fed multilevel, multivariate regression models, with either WHZ_−2_ or MUAC_125_ as the dependent variable and age, sex, stunting, and ‘longer legs’ as the independent ones. We also compared how the performance of MUAC_125_ and WHZ_−2_ to detect slim children, i.e. children with a low Weight-for-Age (WAZ<−2) but no linear growth retardation (HAZ≥−2), was modified by the contributors.

**Results:**

Overall 23.1 % of the 14,409 children were identified as acutely malnourished by either WHZ_−2_ or MUAC_125_, but only 28.5 % of those (949/3,328) were identified by both indicators. Being stunted (+17.8 %; 95 % CI: 14.8 %; 22.8 %), being a female (+16.5 %; 95 % CI: 13.5 %; 19.5 %) and being younger than 24 months (+33.6 %; 95 % CI: 30.4 %; 36.7 %) were factors strongly associated with being detected as malnourished by MUAC_125_ and not by WHZ_−2_, whereas having ‘longer legs’ moderately increased the diagnosis by WHZ_−2_ (+4.2 %; 95 % CI: 0.7 %; 7.6 %). The sensitivity to detect slim children by MUAC_125_ was 31.0 % (95 % CI: 26.8 %; 35.2 %) whereas it was 70.6 % (95 % CI: 65.4 %; 75.9 %) for WHZ_−2_. The sensitivity of MUAC_125_ was particularly affected by age (57.4 % vs. 18.1 % in children aged < 24 months vs. ≥ 24 months). Specificity was high for both indicators.

**Conclusions:**

MUAC_125_ should not be used as a stand-alone criterion of acute malnutrition given its strong association with age, sex and stunting, and its low sensitivity to detect slim children. Having ‘longer legs’ moderately increases the diagnosis of acute malnutrition by WHZ_−2_. Prospective studies are urgently needed to elucidate the clinical and physiological outcomes of the various anthropometric indicators of malnutrition.

## Background

According to WHO experts, a weight-for-height Z-score lower than −3 standard deviations of the international reference population WHO 2006 (WHZ_−3_) or a mid-upper arm circumference lower than 115 mm (MUAC_115_) can be used independently to indicate severe acute malnutrition (SAM) [1]. The commonly used thresholds for global acute malnutrition (GAM) are WHZ<−2 (WHZ_−2_) or MUAC < 125 mm (MUAC_125_). However, these two indicators correlate poorly. It was reported that only about 40 % of SAM cases identified by one indicator are also diagnosed as such by the other [1]. For example, among severely malnourished children hospitalized in rural Kenya, 65.1 % (486/746) of the WHZ_−3_ cases also had a MUAC < 115 mm, whereas 56 % (489/873) of the MUAC_115_ cases were also identified by WHZ_−3_ [2]. In that study, 42.9 % (489/1140) of the SAM cases were identified by both indicators. The discrepancy between the two indicators can be even more extreme [3–5]. Fernandez et al. reported that among 34,937 children between the ages of 6 and 59 months from 39 nutritional surveys, 75 % of the children with a WHZ<−3 were not identified by a MUAC < 115 mm [3]. In Cambodia, this proportion was above 90 %, whereas 80 % of MUAC_115_ were not detected by WHZ_−3_ [4].

Such discrepancy generates important programmatic challenges and confusion [6]. On the one hand, a strategy where the diagnosis can be based on either indicator, as recommended by some authors [1, 4] may unduly inflate the workload of nutritional rehabilitation programmes, as the most appropriate management of children identified by one indicator and not by the other is uncertain. On the other hand, relying on only one of these indicators, e.g. using only MUAC_115_ in community-based programmes, may under-detect true acute malnutrition cases and result in missed opportunities to treat a severe condition [4, 7].

Thus, it is of paramount importance to gain more insight into the factors contributing to this diagnostic discrepancy in order to guide programmatic decisions.

These factors could be three-fold. First, the diagnosis of acute malnutrition based on MUAC relies on the utilization of a single cut-off independently of age and sex. These are factors known to be strongly associated with child anthropometry. As the reference MUAC is lower in younger children (< 24 months) and in females [8], a single cut-off to define acute malnutrition may result in the over-diagnosis of the condition in these sub-populations, and conversely may underestimate cases in older children, particularly in males [2, 9]. Diagnosis based on WHZ would be much less sensitive to this bias because it is standardized for sex and height, and thus indirectly for age [2, 9].

Second, the diagnosis of acute malnutrition by MUAC may be confounded by the presence of stunting given the strong association between MUAC and height-for-age Z-score (HAZ); using MUAC_125_ would result in an increased caseload among stunted children compared to non-stunted children, independently of acute malnutrition [2, 10].

Third, WHZ may overestimate acute malnutrition in children with a low sitting-to-standing height ratio (SSR), i.e. with long limbs, whereas MUAC would be relatively independent of body proportions [11]. Ethnic differences in SSR, like those observed between nomads and settled populations, or between African and Asian populations, might thus influence the diagnosis by WHZ, without indicating acute malnutrition. If this hypothesis is true, the same variations should be also observed within a given population, i.e. the individuals with longer legs should be more often identified as acutely malnourished by WHZ_−2_ than individuals with shorter legs.

The objective of this study is to test simultaneously these three hypotheses in a large multi-ethnic population, and to assess the contribution of age, sex, stunting, and SSR to the discrepant diagnosis of acute malnutrition by either WHZ_−2_ or MUAC_125_.

## Methods

We analyzed data from 16 cross-sectional nutritional surveys conducted by Action Against Hunger International in South Sudan (13 surveys), the Philippines (1 survey), Chad (1 survey), and Bangladesh (1 survey). The surveys in South Sudan were conducted from 2004 to 2007 in different counties of the Upper Nile, Western Equatoria, Eastern Equatoria, and Bahr El Ghazal regions, mainly amongst agro-pastoralist communities of varying ethnic background. The survey in the Philippines was conducted in 2012 in the city of Cagayan de Oro. The survey in Chad was conducted in 2013 in the North Bahr El Ghazal district, among the Gorane pastoralist, nomadic population. All these surveys followed a two-stage cluster representative sampling methodology. In Bangladesh, data were collected in November 2012, through the regular monthly Growth Monitoring Program for children under 5 years old living in the Rohynguas refugee camps of Kutapalong and Nayapara in Cox’s Bazaar district.

Procedures to measure the sitting height were similar to those described in previous studies [11, 12]. Crown-rump length, a measure of trunk length, which is conceptually similar to sitting height in older children, was measured until the age of 2 years. Children laid down in supine position on the length board, and after the thighs were placed in a vertical plane, the footboard was pulled against the buttocks. From 2 years of age onward, sitting height was measured with the child sitting in erect position on a flat wooden table, with the knees at the edge of the table and the buttocks against the height board. Weight (with a SECA electronic scale or a SALTER scale of 25 kg), length or height (with locally made wooden boards), and MUAC (with a no stretchable MUAC band) were measured following the international recommendations, to the nearest 100 g or 1 mm, respectively. To determine age (months), the birth date was either extracted from official documents (e.g. birth certificates, refugee registers) or evaluated by maternal report in reference to locally adapted seasonal calendars when official documents were unavailable.

Individual Z-scores for WHZ and HAZ were computed in reference to the WHO 2006 growth standards using the Stata zscore06 command. We computed SSR as (Sitting height/standing height)*100 and generated individual Z-scores for the SSR within strata of 6 months of age of the pooled study population (SSRZ), as SSR is largely age-dependent and no reference distribution exists for SSR [12].

The initial dataset included 14,682 individuals. Cases of edematous malnutrition were excluded from analysis because the WHZ indicator is not valid in such cases (*n* = 15). We also excluded 258 outliers. These were defined based on the WHO standards with recommended flag limits: WHZ-5, +5; HAZ-6, +6 (source: http://www.who.int/nutgrowthdb/software/Differences_NCHS_WHO.pdf?ua=1). For SSRZ, as no reference population exists, we used the flag limits −3 SD, +3SD of the study population. Therefore, the final dataset included 14,409 children. Dependent variables were defined as either WHZ_−2_ or MUAC_125_. Independent variables were dichotomized to correspond to categories usually used in field nutrition programs: young age (age < 24 months vs. age ≥ 24 months), stunting (HAZ<−2SD vs. HAZ≥−2SD of the reference population), sex (male vs. female). We also created the variable ‘longer legs’ by dichotomizing SSRZ (SSRZ in the lower tertile vs. SSRZ in the 2 upper tertiles of the study population). These independent variables correspond with hypothetical contributors to the discrepant malnutrition diagnosis presented in the background section.

We tested the association of these independent variables with either WHZ_−2_ or MUAC_125_ by running multivariate multilevel linear regression models with ‘survey’ as a random effect to account for the clustering of data. In linear regression models, where both dependent and independent variables are dichotomous, the regression coefficient corresponds to the absolute percentage difference in the dependent variable (e.g. MUAC_125_) between the categories of the independent variable (e.g. males vs. females) [13]. In such models, linearity is always ensured and results are mathematically equivalent to those of a chi-square test, whereas adjustment for covariates and clustering of data is made possible. All statistical models included all covariates (i.e. sex, young age, stunting and ‘longer legs’) to control for potential confounding effects.

To assess the discrepancy between WHZ_−2_ and MUAC_125_ more specifically, we conducted the same analysis described above with a dummy variable coded 1 for cases of acute malnutrition identified by MUAC_125only_ (MUAC < 125 mm and WHZ>−2) and coded 0 for cases identified by WHZ_-2only_ (WHZ<−2 and MUAC > 125 mm). Finally, we compared the performance of MUAC_125_ and WHZ_−2_ to detect slim children, i.e. children too light for their age (WAZ<−2SD), yet not stunted (HAZ≥−2SD). The purpose of this exploratory analysis was to assess how sex, age, stunting and body proportions modify the sensitivity and specificity of MUAC_125_ and WHZ_−2_ to identify such cases. For this purpose, we ran the same models as described above, with “slimness” as the independent variable and sex, age, and ‘longer legs’ as covariates. The sensitivity of MUAC_125_ or WHZ_−2_ to detect slimness with adjustment for covariates and study design can be derived from such regression models by summing up the regression coefficient of the independent variable (“slimness”) with the constant of the model. Adjusted specificity is computed in the same way by inserting the reciprocal of the dependent and independent variables in the regression models. Interactions between covariates and “slimness” were tested using a chunk test, i.e. a likelihood ratio test comparing the models with and without the full set of interaction terms [14]. When the chunk test was statistically significant, we computed sensitivity and specificity by strata of covariates (age, sex, and ‘longer legs’), with adjustment for other covariates and clustering of data.

Outliers were excluded from the main analysis, but their influence on results was tested by a sensitivity analysis. Significance level was set at 5 % for all tests. All analyses were performed in Stata 12.0 (College Station, TX77845, USA).

As data came from past surveys carried out by Action Against Hunger International in the frame of their nutritional programs and were anonymous, no ethical clearance was requested.

## Results

A sample of 14,682 children aged 6–59 months were included. After removal of 15 cases of edematous malnutrition and 258 outliers, 14,409 individuals (> 98 %) contributed to further analysis. The mean (±SD) age of participants was 31.9 (±15.8) months, with 34.8 % being younger than 24 months. Most of the children (70.9 %) were from an African setting (South Sudan or Chad).

Overall 23.1 % of children were identified as acutely malnourished by either WHZ_−2_ or MUAC_125_, with a prevalence close to 31 % in the children aged less than 24 months (Table 1). Figures returned by these two indicators varied overall and by age range. Acute malnutrition defined by MUAC_125_ was overall present in 10.8 % (1,550/14,409) of children, with 70.6 % (1,094/1,550) of the cases younger than 24 months whereas acute malnutrition as defined by WHZ_−2_ was present in 18.9 % (2,727/14,409) of children, with 41.3 % (1,127/2,727) of the cases younger than 24 months. The proportion of congruent diagnosis returned by the two indicators was only 28.5 % (949 of the 3,328 children identified by either WHZ_−2_ or MUAC_125_) (Table 1). This proportion was highly variable, even within the same country, ranging from 19.8 % in Sudan-Myaiendit to 39.7 % in Sudan-Padak.

**Table 1:** Characteristics of study population

	N	Male %	Age < 24 months %	Stunted %	SSR mean	MUAC_125_ %	WHZ_−2_ %	MUAC_125_ or WHZ_−2_ %	MUAC_125_ and WHZ_−2_ %	Overlap between 2 indicators %
Survey										
Bangladesh	3,831	50.5	32.2	59.3	58.6	8.5	15.0	18.8	4.7	25.0
Chad	718	51.1	34.1	13.5	56.2	6.8	34.1	35.2	5.7	16.2
Philippines	368	50.0	8.7	38.6	55.7	0.5	3.0	3.0	0.5	18.2
Sudan-Myaiendit	465	51.4	35.7	10.5	55.9	8.2	20.4	23.9	4.7	19.8
Sudan-Renk	613	49.4	42.9	13.9	56.1	11.4	17.6	21.2	7.8	36.9
Sudan-Malut	705	47.2	38.0	15.3	56.5	4.4	21.1	21.7	3.8	17.6
Sudan-Kapoetat	749	48.1	33.0	28.0	57.1	11.2	10.8	16.6	5.5	33.1
Sudan-Padak	906	48.5	43.0	9.7	55.5	28.9	42.9	51.4	20.4	39.7
Sudan-Malakal	751	48.1	41.8	7.7	56.6	7.9	21.6	23.6	5.9	24.9
Sudan-Kajokeji	901	48.6	37.1	22.6	57.4	6.7	9.0	11.4	4.2	36.9
Sudan-Nyadintoch	774	47.8	34.0	18.9	54.5	18.2	21.2	30.1	9.3	30.9
Sudan-Mareang	748	47.3	33.0	18.9	54.4	18.3	21.4	30.2	9.5	31.4
Sudan-Mvolo	658	54.0	30.2	24.5	55.9	7.0	8.4	11.2	4.1	36.5
Sudan-Oldfangak	909	51.9	35.4	5.5	55.6	9.4	18.5	22.2	5.6	25.2
Sudan-Bunagok	420	49.5	38.6	16.0	56.1	10.7	12.9	17.4	6.2	35.6
Sudan-Duk	893	53.3	36.4	15.9	55.8	12.8	25.9	30.3	8.3	27.3
Age										
< 24 months	5,010	50.2	/	25.1	59.8	21.8	22.5	31.1	13.3	42.7
≥ 24 months	9,399	49.7	/	29.4	55.0	4.9	17.0	18.9	3.0	16.0
Sex										
Female	7,217	/	34.6	25.6	56.6	11.9	17.3	22.7	6.5	28.7
Male	7,192	/	35.0	30.2	56.8	9.6	20.5	23.5	6.6	28.3
Stunting										
No	10,388	48.3	36.1	/	56.2	9.5	19.5	23.0	6.0	26.0
Yes	4,021	54.0	31.2	/	58.0	14.1	17.5	23.5	8.2	34.9
Long leg										
No	9,692	51.1	37.8	37.3	58.3	9.8	15.0	19.0	5.8	30.3
Yes	4,717	47.5	28.5	8.6	53.3	12.8	27.0	31.5	8.3	26.3
Total	14,409	49.9	34.8	27.9	56.7	10.8	18.9	23.1	6.6	28.5

*MUAC*_*125*_ Mid upper arm circumference < 125 mm, *WHZ*_*−2*_ Weight-for-height Zscore<−2, *SSR* Sitting-standing height ratio (sitting height/standing height) × 100, *Long leg* SSR Zscore in the lower tertile of the study population, *Stunting* Height-for-age Zscore<−2, *Overlap* Proportion of cases being MUAC_125_ and WHZ_−2_ among children identified by either MUAC_125_ or WHZ_−2_

Having ‘longer legs’, being stunted, being a male and being younger than 2 years were all factors independently associated with acute malnutrition as diagnosed either by WHZ_−2_ or MUAC_125_ (Table 2). However, the strength of the association differed by anthropometric indicator. The diagnosis by WHZ_−2_ was +10.6 % (95 % CI: +9.1 %; +12.1 %) more frequent in children with ‘longer legs’, yet only +3.4 % (95 % CI: +2.3 %; +4.6 %) more frequent for MUAC_125_. On the contrary, being stunted and of young age were factors much less associated with WHZ_−2_ than with MUAC_125._ The diagnosis by MUAC_125_ was +9.2 % (95 % CI: +8.0 %; +10.4 %) higher in stunted children than in non-stunted children, and +17.1 % (95 % CI: +16.1 %; +18.2 %) higher in children younger than 24 months than in older ones; the corresponding increase was 3.4 % and 5.6 % for WHZ_−2_, respectively_._ With regards to sex, the relationship was the opposite for WHZ_−2_ (+3.4 % in males) compared to MUAC_125_ (−2.6 % in males).

**Table 2:** Factors associated with the diagnosis of acute malnutrition based on WHZ_−2_ and MUAC_125_

Indicator	WHZ_−2_ (*n* = 2 727)				MUAC_125_ (*n* = 1 550)				MUAC_125only_ (*n* = 601)			
Population	All (*N* = 14 409)				All (*N* = 14 409)				MUAC_125only_ OR WHZ_-2only_ (*N* = 1 778)			
Independent variable	β coeff.	95 % CI		*P*	β coeff.	95 % CI		*P*	β coeff.	95 % CI		*P*
Long legs (yes vs. No)	10.6	9.1	12.1	< 0.0001	3.4	2.3	4.6	< 0.0001	−4.2	−7.6	−0.7	0.019
Stunted (yes vs. No)	4.1	2.5	5.6	< 0.0001	9.2	8.0	10.4	< 0.0001	18.8	14.8	22.8	< 0.0001
Sex (male vs. female)	3.4	2.1	4.6	< 0.0001	−2.6	−3.5	−1.6	< 0.0001	−16.5	−19.5	−13.5	< 0.0001
Age (< 24 months vs. ≥ 24 months)	5.6	4.3	7.0	< 0.0001	17.1	16.1	18.2	< 0.0001	33.6	30.4	36.7	< 0.0001

Results are derived from multivariate multi-level linear regression models, with “survey” as random effect. The regression coefficients represent the absolute % increase in the dependent variables (WHZ_−2_, MUAC_125,_ MUAC_125_) between the 2 categories of the independent variables (long legs, stunted, sex, age). All models were adjusted for all covariates

The analysis of being detected by MUAC_125only_ vs. WHZ_-2only_ returned statistical associations consistent with those described above. Being stunted (+18.8 %), being a female (+16.5 %) and young age (+33.6 %) were factors strongly associated with being detected as malnourished by MUAC_125only_, whereas having ‘longer legs’ increased moderately the diagnosis by WHZ_-2only_ (+4.2 %; 95 % CI: +0.7 %; +7.6 %).

Among the 10,388 children who were not stunted, 13.3 % (1,383/10,388) were considered slim, i.e. they had a low weight-for-age (WAZ<−2). Overall, the sensitivity to detect slim children by WHZ_−2_ (70.6 %; 95 % CI: 65.4 %; 75.9 %) was substantially higher than by MUAC_125_ (31.0 %; 95 % CI: 26.8 %; 35.2 %), whereas the specificity was very high for both indicators. The chunk test was highly significant for both indicators, and all covariates had a modifying effect on the sensitivity and specificity although the size of this modification varied across covariates and indicators (Table 3). The sensitivity of MUAC_125_ to detect slim children was lower in males than in females and much lower after 24 months of age than in younger children (18.1 % vs. 57.4 %). However, the difference between children with ‘longer legs’ and the others was negligible. For WHZ_−2_, the largest difference in sensitivity was observed between children with ‘longer legs’ compared to those without (84.2 % vs. 68.3 %). The sensitivity of WHZ_−2_ to detect slim children remain much higher than that of MUAC_125_ in children who did not have ‘longer legs’. Although the specificity was globally high for both indicators, the specificity of MUAC_125_ decreased in children younger than 24 months (87.7 %), and the specificity of WHZ_−2_ decreased in children with ‘longer legs’ (82.9 %).

**Table 3:** Sensitivity and specificity of MUAC_125_ and WHZ_−2_ to detect slim children

		Total N	Slim n	WHZ_−2_ (*n* = 2 022)						MUAC_125_ (*n* = 983)					
Covariates				Sensitivity	95 % CI		Specificity	95 % CI		Sensitivity	95 % CI		Specificity	95 % CI	
Long legs	Yes	4,309	847	84.2	75.9	92.5	82.9	73.7	92.1	32.2	26.4	38.0	96.8	90.2	100
	No	6,079	536	68.3	63.9	72.8	95.2	90.2	100	32.0	28.0	36.1	97.5	92.9	100
Sex	Male	5,020	720	73.4	67.4	79.3	93.6	86.9	100	25.9	20.9	30.9	99.2	93.7	100
	Female	5,368	663	68.8	62.7	75.0	93.4	86.6	100	33.6	28.7	38.6	100	94.8	100
Age < 24 months	Yes	3,754	664	75.0	69.9	80.2	95.0	89.2	100	57.4	51.1	63.7	87.7	80.6	94.8
	No	6,634	719	65.8	59.1	72.5	94.1	86.9	100	18.1	14.0	22.1	97.6	93.1	100
Total		10,388	1,383	70.6	65.4	75.9	93.9	88.3	99.6	31.0	26.8	35.2	98.4	93.9	100

Slimness was defined as a WAZ<−2 in children with no linear growth retardation, i.e. with HAZ≥−2. Adjusted sensitivity and specificity are derived from multivariate multi-level linear regression models, with “survey” as random effect. The regression coefficients represent the absolute % in the dependent variable (WHZ-2 or MUAC125) between the categories of the independent variable (slimness). Sensitivity is computed by summing the regression coefficient with the constant of the regression model. Specificity is computed by using the reciprocal of the dependent and independent variables in the model. All models were adjusted for all covariates. Interactions between covariates and slimness were tested all together through a chunk test. The chunk test was highly significant for both indicators, and all covariates had a modifying effect on the sensitivity and specificity

Among the 1,383 slim children, 80.5 % (1,113/1,383) were detected by either WHZ_−2_ or MUAC_125,_ but only 32.6 % (451/1,383) were identified by both indicators. Of the 508 cases identified by MUAC_125_, 88.8 % were also identified by WHZ_−2_, whereas among cases undetected by MUAC_125_ (*n* = 875), the diagnosis by WHZ_−2_ was consistent in only 30.9 %. Conversely, in cases undetected by WHZ_−2_, the diagnosis by MUAC_125_ was consistent in 82.6 %.

Including outliers in the analysis did not modify substantially the results (changes in coefficients were all < 5 %).

## Discussion

The diagnosis of acute malnutrition by either MUAC_125_ or WHZ_−2_ was globally congruent in less than one third of the cases diagnosed in this multi-survey population, and the proportion of congruence was variable between surveys. Being a female, less than 24 months of age, and stunted were strong predictors of MUAC_125,_ whereas having ‘longer legs’ was a factor associated with a moderate increase in WHZ_−2_. The sensitivity of MUAC_125_ for slimness (as defined by WAZ<−2 and HAZ≥−2) was overall low and extremely low in children aged ≥ 24 months. The specificity of both indicators for slimness was overall high, except in children with ‘longer legs’ for WHZ_−2_ (82.9 %) and in young children for MUAC_125_ (87.7 %).

The prevalence of MUAC_125_ was much lower in children aged ≥ 24 months than in younger ones, whereas the difference in the proportion of WHZ_−2_ between these two age categories was much smaller. Two explanations can be proposed. First, as MUAC_125_ relies on a single cut-off from age 6 to 59 months, it does not account for the higher MUAC observed in older normal children. Therefore, the sensitivity of MUAC_125_ is expected to drop with increasing age, making it a poor screening tool for acute malnutrition after age 24 months. Our results support that explanation. The sensitivity of MUAC_125_ to detect slim children was 57.4 % in children under 24 months of age, but dropped to 18.1 % in older children. The difference in sensitivity by age was also observed for WHZ_-2,_ but was much smaller (75.0 % vs. 65.8 %). The overall congruence between the 2 indicators increased to 42.2 % in children with age ≤ 24 months. Our results are consistent with those reported by Myatt in a review of more than 500 surveys [15].

An alternative hypothesis is that WHZ_−2_ would exaggerate the diagnosis of acute malnutrition in populations with ‘longer legs’ (low SSR). In our results the specificity of WHZ_−2_ to identify slim children indeed decreased to 82.9 % in children with ‘longer legs’, whereas the specificity of MUAC_125_ was more robust. However, the contribution of SSR to the diagnosis discrepancy between the 2 indicators was small, with a congruence ranging from 26.3 % in children with ‘longer legs’ to 29.7 % in others. The multivariate analysis of being detected by MUAC_125only_ vs. WHZ_-2only_ showed that having ‘longer legs’ resulted in a moderate increase of WHZ_-2only_ by about +4 %. Moreover, we observed that MUAC_125_ also increased in children with ‘longer legs’ in multivariate analysis, although less strongly than WHZ_-2._ A previous study carried out in a sample of children aged 24 to 59 months in Ethiopia reported also that both WHZ and MUAC were associated with SSR, with a weaker association for MUAC than for WHZ [11]. The implications in terms of clinical management and outcome are unknown, and should be investigated in further studies.

Being stunted was a factor associated with both indicators, but more strongly with MUAC_125_ than with WHZ_−2_. In our analysis, 18.8 % more children were identified by MUAC_125only_ if they were stunted. Stunting is usually considered to result from a history of sub-optimal nutrition and poor health. It is therefore not unexpected that stunted children be also more vulnerable to episodes of acute malnutrition. The stronger association of stunting with MUAC_125_ than with WHZ_−2_ is less straightforward. MUAC is seen by some authors as mainly a measure of muscle mass [16–18] and muscle mass is reduced in stunted children [17]. Consistently, a study in Kenya reported that upper arm muscle area Z-score, an age and gender adjusted measure of absolute lean body mass, explained most of the variability in the progression of stunting among more than 1,000 school-aged children over a 2 year-period [19]. Therefore, a low MUAC is also an indicator of stunting, in contrast to WHZ which is standardized for sex and height. This could also explain why the overlap of MUAC_125_ and WHZ_−2_ was greater in stunted than in not stunted children in our study, as a proportion of children with a WHZ_−2_ in stunted children would also be identified by MUAC_125_ because of the presence of stunting. The extent to which acute malnutrition could be overestimated by MUAC_125_ in stunted children or underestimated in not stunted children remains to be elucidated. For example, a study in Burkina Faso where children were admitted for nutritional rehabilitation on the basis of a low MUAC only reported that the mean daily gain in MUAC and weight was lower, the treatment duration longer and the proportion of non-responders greater in smaller children [20]. This might correspond to a suboptimal response in less severely acutely malnourished children, or be an indicator that the treatment is less effective or required in such children. It is worth mentioning that the same observation was also reported for younger children and in females in that study, i.e. in children presenting characteristics independently associated with a low MUAC.

On the other hand, the extent to which the association of stunting and low MUAC would increase the severity of the pathophysiological profile of acute malnutrition in the affected children also needs to be carefully examined. A recent meta-analysis showed a dramatic increase in mortality risk in children combining low WHZ and stunting [21]. A similar analysis is needed for MUAC.

Our study is the first one to document the concomitant influences of age, sex, HAZ and ‘longer legs’ on the diagnosis of acute malnutrition by either MUAC_125_ or WHZ_-2._ However, it is not without limitations. First, we used routine data collected in a growth monitoring program or data collected in field surveys conditions, which may have an impact on the accuracy of measurements. However, assessors received a training or a refresher session prior to the data collection, and outliers were few. Second, the analysis was based on cross-sectional assessments of anthropometric indicators alone with no possibility to assess the functional outcomes of the various malnutrition indicators. While this was useful to examine the pre-existing hypotheses to explain diagnostic discrepancy, we believe that further investigations are required now to formally analyze the pathophysiology and functional severity of the cases diagnosed by the different types of anthropometric deficits. For example, there are indications that children presenting with both MUAC_125_ and WHZ_−2_ [2] or with both stunting and a low WHZ [21] are at higher risk of dying.

Today there is insufficient evidence to recommend the use of one unique indicator of acute malnutrition. The low sensitivity of MUAC_125_, even in children ≤ 24 months, is particularly worrisome if applied as the unique indicator in program screening. Consistently, a recent study reported that using MUAC alone to identify severely malnourished children for admission in a therapeutic centre would have failed to identify 33 % of deaths, while 98 % were identified by WHZ<−3 alone [7]. Prospective studies on the clinical and physiological outcomes of the various anthropometric indicators of malnutrition are urgently needed.

## Abbreviations

HAZ: Height-for-age Z-score
MUAC_125_: Mid upper arm circumference < 125 mm
SSRZ: Sitting-standing height ratio Z-score
WAZ: Weight-for-age Z-score
WHZ-2: Weight-for-height Z-score<−2
